# Predictive Accuracy of Cord Blood C-reactive Protein and Immature-to-Total Neutrophil Ratio for Early-Onset Neonatal Sepsis in High-Risk Deliveries: A Prospective Observational Study at a Tertiary Care Center in Central India

**DOI:** 10.7759/cureus.102752

**Published:** 2026-01-31

**Authors:** Parul Dohar, Manjusha Goel, Neha Shrivastava, Aapurti Awasthi, P. Amrutha

**Affiliations:** 1 Pediatrics, Gandhi Medical College, Bhopal, Bhopal, IND

**Keywords:** biomarkers, blood culture, cord blood, c-reactive protein, crp, early-onset neonatal sepsis, india, i/t ratio, newborn infection, predictive accuracy

## Abstract

Background

Early-onset neonatal sepsis (EOS) remains a critical contributor to neonatal mortality, particularly in low- and middle-income countries like India. Blood culture, while considered the diagnostic gold standard, is often limited by low sensitivity and delayed results. Cord blood biomarkers such as C-reactive protein (CRP) and the immature-to-total neutrophil ratio (I/T) offer promising alternatives for early, non-invasive detection of EOS.

Methods

This prospective observational study was conducted at a tertiary-care teaching hospital in Central India between June 2023 and December 2024. A total of 170 neonates born to mothers with one or more risk factors for EOS were enrolled. Umbilical cord blood and peripheral venous blood were analyzed for CRP, I/T ratio, complete blood count (CBC), and culture. Receiver operating characteristic (ROC) analysis was used to determine diagnostic performance and optimal cutoff values.

Results

Cord blood culture was positive in 25.9% of cases, with coagulase-negative *Staphylococcus aureus* being the most frequent isolate. Cord blood CRP ≥3.3 mg/dL and I/T ratio ≥0.155 were significantly associated with sepsis and demonstrated excellent diagnostic performance (area under the curve (AUC): 1.000 and 0.878, respectively). Comparable markers in venous blood also showed high sensitivity but slightly reduced specificity. Strong concordance was observed between cord and venous blood culture results (p<0.001).

Conclusion

Cord blood CRP and I/T ratio are reliable, immediate, and non-invasive markers for identifying EOS in high-risk neonates. Their inclusion in early screening protocols could significantly reduce diagnostic delays and unnecessary antibiotic exposure.

## Introduction

Early-onset neonatal sepsis (EOS), defined as infection within the first 72 hours of life, remains a leading cause of neonatal morbidity and mortality worldwide [1]. In high-income countries, its incidence ranges from 1 to 4 per 1,000 live births, whereas South Asian and Sub-Saharan settings report rates up to 15-20 per 1,000, with over 600,000 neonatal deaths annually attributed to sepsis as of 2019 [2]. In India alone, clinical EOS incidence reaches approximately 17,000 per 100,000 live births, equating to nearly 17% of newborns [3]. Despite significant improvements in perinatal care, high case fatality rates underscore the urgent need for reliable early diagnostic strategies [4]. Timely detection of EOS is challenging. Blood culture, considered the diagnostic gold standard, often yields false-negative results in up to 50% of cases owing to low sample volume and prior maternal antibiotic exposure, and requires 48-72 hours for pathogen confirmation. Moreover, conventional biomarkers such as C-reactive protein (CRP) and procalcitonin (PCT) may demonstrate delayed elevation depending on the timing of sampling, gestational age, and clinical context, and often lack sufficient specificity, leading to widespread empirical antibiotic use in neonates; this overuse contributes to microbiome disturbances and antimicrobial resistance [5].

Umbilical cord blood offers a uniquely advantageous matrix for immediate, non-invasive screening of EOS. Several inflammatory cytokines, including IL-6, IL-8, IL-1β, and IL-18, are elevated in cord blood among neonates who develop early-onset sepsis [6], with IL-6 and IL-8 demonstrating particular diagnostic potential, especially in preterm infants [7,8]. In addition, proteomic studies have identified multiple acute-phase reactants in cord plasma, such as CRP, serum amyloid A (SAA1), and lipopolysaccharide-binding protein (LBP), further supporting early immune activation at birth [9]. Although these findings support the potential role of cord blood biomarkers for early risk stratification, existing evidence is predominantly derived from small, single-center studies conducted in controlled or well-resourced settings, with limited data reflecting real-world implementation in resource-constrained healthcare systems.

The primary objective of this study was to evaluate the diagnostic accuracy of umbilical cord blood CRP and immature-to-total neutrophil (I/T) ratio for predicting culture-proven EOS. Secondary objectives included assessment of other cord blood hematological parameters, comparison with corresponding venous blood biomarkers, and evaluation of concordance between cord and venous blood culture results. Conducted at a tertiary-care center in India, this study leverages the non-invasive, routinely available nature of cord blood sampling to assess its role in early sepsis prediction. CRP and hematologic indices, reflecting early inflammatory response, were selected for their accessibility and biological relevance. We hypothesize that abnormalities in these parameters at birth, such as elevated CRP, leucocytosis, or left shift, will correlate with subsequent sepsis diagnosis and can serve as early indicators to guide clinical decision-making. Accordingly, this study aims to improve the early detection of EOS, facilitate timely and appropriate initiation of treatment, minimize unnecessary empirical antibiotic exposure, and support targeted, evidence-based clinical management.

## Materials and methods

This was a hospital-based prospective observational study conducted with the objective of evaluating the predictive value of umbilical cord blood biomarkers, specifically CRP, complete blood count (CBC) parameters, and blood culture in identifying EOS. The study was carried out in the Department of Pediatrics, Gandhi Medical College and Associated Kamla Nehru Hospital, Bhopal, which serves as a tertiary-care referral center in Central India. The data collection spanned an 18-month period, from June 2023 to December 2024. The study population included hospital-born neonates delivered to mothers with one or more risk factors for EOS. These risk factors included spontaneous prematurity, prolonged rupture of membranes (>24 hours), foul-smelling liquor, prenatal fever, prenatal laboratory-confirmed septicaemia, chorioamnionitis, maternal urinary or genital tract infections, multiple vaginal examinations during labor, and prolonged labor (>24 hours). Neonates with a gestational age below 28 weeks or those with lethal congenital anomalies were excluded. Gestational age was assessed using first-trimester ultrasonography when available, or by last menstrual period, corroborated with postnatal New Ballard scoring. Neonates born before 28 weeks of gestation were excluded due to substantially different inflammatory responses and clinical management patterns in extremely preterm infants, which could confound biomarker-based diagnostic accuracy. Maternal risk factors were documented individually for each participant and used primarily as eligibility criteria for study inclusion, while descriptive and inferential analyses focused on neonatal biomarker performance rather than cumulative maternal risk scoring.

Sample size calculation was based on the formula for estimating proportions: $n = \frac{Z^2 \times p \times q}{d^2}$ where Z is the standard normal variate at 95% confidence level (1.96), p is the estimated prevalence of neonatal sepsis (1.8%), q = 100-p = 98.2%, and d is the margin of error (2%). Substituting the values, the calculated sample size came out to be 170 neonates. The prevalence estimate was selected to reflect culture-confirmed EOS rather than clinically suspected sepsis, thereby providing a conservative and methodologically appropriate estimate for diagnostic accuracy evaluation. All eligible neonates delivered during the study period were consecutively screened and approached for enrollment. Enrollment was dependent on parental consent. No systematic exclusion of eligible neonates occurred, thereby reducing the potential for selection bias. A pre-tested semi-structured questionnaire was used as the data collection tool, as given in the Appendices. Data collectors underwent structured training on standardized data collection procedures prior to study initiation. Periodic supervision and cross-verification by the principal investigator were performed to ensure consistency and minimize inter-observer variability. The tool captured maternal risk factors, perinatal history, gestational age, birth weight, Apgar scores, clinical signs of sepsis, and laboratory findings. The data collection proforma used in this study was pre-tested on a small subset of eligible neonates prior to the commencement of formal data collection. The pre-testing aimed to assess the clarity, relevance, and feasibility of the tool in the clinical setting. Based on the feedback obtained, necessary modifications were incorporated to improve the comprehensiveness and user-friendliness of the questionnaire.

Immediately after birth, and prior to placental transfusion, the umbilical cord was clamped using sterile technique. Delayed cord clamping was not performed in study participants to ensure uniformity of cord blood sampling and to minimize variability in biomarker concentrations. Cord blood was subsequently collected from the placental end under aseptic conditions. A 5 mL cord blood sample was collected from the placental end of the umbilical vein using a sterile 22-gauge syringe after disinfecting the cord surface at the sampling site with 70% isopropyl alcohol to minimize contamination. Of this, 2 mL was transferred into blood culture bottles under aseptic conditions and sent to the microbiology laboratory without delay. The remaining 3 mL was divided into one portion transferred into an ethylenediaminetetraacetic acid (EDTA) vial for CBC analysis using an automated hematology analyzer, and the other into a plain vial for CRP measurement via a fully automated immunoturbidimetric analyzer. Laboratory equipment was calibrated daily using manufacturer-provided standards to ensure accuracy of results. All neonates were admitted to the NICU for close monitoring. Venous blood sampling at 24-48 hours was performed prior to antibiotic initiation whenever clinically feasible. Empirical antibiotics were started according to the Facility-Based Newborn Care (FBNC) guidelines, with immediate initiation reserved for clinically unstable neonates, after obtaining blood samples wherever possible. Data collectors included trained pediatric residents and neonatal intensive care unit (NICU) nurses, who were sensitized to the study protocol and underwent structured training prior to study initiation to ensure uniformity in sample collection and documentation. To ensure data quality and confidentiality, all records were anonymized and entered into password-protected spreadsheets. The senior investigator cross-checked 20% of the data entry to minimize entry errors.

Data was entered in Microsoft Excel (version 16.105.1; Microsoft Corp., Redmond, WA, USA) and analyzed using IBM SPSS Statistics for Windows, Version 20 (Released 2011; IBM Corp., Armonk, New York, United States). Continuous variables were summarized as mean and standard deviation (SD), while categorical variables were expressed as frequencies and percentages. The distribution of continuous variables was assessed for normality using Shapiro Wilk test prior to applying appropriate inferential tests. Group comparisons for continuous variables between neonates with positive and negative blood culture results were performed using the Mann-Whitney U test. Categorical data was analysed using the chi-square test. Receiver operating characteristic (ROC) curve analysis was employed to assess the diagnostic accuracy of cord and venous blood biomarkers in predicting EOS. Area under the curve (AUC), sensitivity, specificity, positive predictive value (PPV), and negative predictive value (NPV) were calculated for each marker. Optimal cutoff values were determined using Youden’s Index. A p-value of <0.05 was considered statistically significant throughout the analysis. The study protocol was approved by the Institutional Ethics Committee of Gandhi Medical College, Bhopal (18850/MC/IEC/2023). Written informed consent was obtained from parents or legal guardians prior to enrollment, in adherence with the Declaration of Helsinki principles. Participants were ensured confidentiality, and no additional risk was imposed as cord blood sampling was done from routinely discarded tissue.

## Results

A total of 170 neonates born to mothers with one or more recognized risk factors for early-onset sepsis were enrolled in the study. Of the 170 cord blood samples analyzed, microbial growth was detected in 44 (25.9%) of cases. Coagulase-negative *Staphylococcus aureus* was the most frequently isolated organism, accounting for 26 (15.3%) of cases, followed by methicillin-sensitive and methicillin-resistant *S. aureus*, each isolated in nine (5.3%) of cases. Venous blood cultures were positive in 35 (20.6%) neonates, demonstrating a broadly similar microbiological profile; however, *Acinetobacter* species were isolated exclusively from venous blood cultures, occurring in eight (4.7%) of cases. Among the maternal risk factors evaluated, prenatal fever was significantly associated with both cord blood culture positivity (p = 0.013) and venous blood culture positivity (p = 0.001), while laboratory-confirmed maternal sepsis showed a strong association with cord blood culture positivity (p < 0.001). Other maternal risk factors, including spontaneous prematurity, foul-smelling liquor, and prolonged rupture of membranes, did not demonstrate statistically significant associations with culture positivity. The baseline maternal and neonatal characteristics, along with the clinical presentation at admission, are summarized in Table 1.

**Table 1:** Baseline Maternal and Neonatal Characteristics and Clinical Presentation at Admission (N = 170)

Parameter	Category	Frequency n (%)
Gestational Age (Weeks)	28-32	24 (14.1)
	32-34	9 (5.3)
	34-36	18 (10.6)
	36-38	94 (55.3)
	>38	25 (14.7)
Gender	Male	86 (50.6)
	Female	84 (49.4)
Birth Weight (kg)	<1.00	16 (9.4)
	1.00-1.49	17 (10.0)
	1.50-2.49	84 (49.4)
	≥2.5	53 (31.2)
Maternal Risk Factors	Spontaneous prematurity	42 (24.7)
	Foul-smelling liquor	8 (4.7)
	Rupture of membranes >24 hours	111 (65.3)
	Prenatal fever	18 (10.6)
	Prenatal laboratory-confirmed sepsis	9 (5.3)
Clinical Presentation at Admission	Dullness/lethargy	35 (20.6)
	Refusal to feed	9 (5.3)
	Respiratory distress	77 (45.3)
	Apnoea	34 (20.0)
	Hypothermia	68 (40.0)
	Hyperthermia	8 (4.7)
	Impaired circulation	45 (25.9)
	Skin pustules/rash	9 (5.3)

When cord blood parameters were compared between neonates with positive and negative venous blood cultures, inflammatory markers demonstrated clear differences between the groups (Table 2). Neonates with positive venous blood cultures had significantly higher cord blood CRP levels (12.21 ± 6.06 mg/dL vs. 3.22 ± 1.25 mg/dL; p < 0.001) and a higher I/T ratio (0.2063 ± 0.0256 vs. 0.1567 ± 0.0293; p < 0.001) compared with culture-negative neonates. In contrast, mean total leukocyte count and platelet count did not differ significantly between the two groups (p = 0.520 and p = 0.889, respectively). The distribution of cord blood absolute neutrophil count (ANC) categories (<1800 vs. ≥1800 cells/mm^3^) was comparable between neonates with positive and negative venous blood cultures, with no statistically significant association observed (χ^2^ = 0.765, p = 0.382).

**Table 2:** Comparison of Cord Blood Parameters Between Neonates With Positive and Negative Venous Blood Cultures (N = 170) ** p < 0.01 indicates statistical significance.

Parameter	Venous Blood Culture	n	Mean ± SD/n (%)	Statistical Test	P-value
C-reactive Protein (CRP) (mg/dL)	Positive	35	12.21 ± 6.06	Mann-Whitney U = 0.0	<0.001**
	Negative	135	3.22 ± 1.25		
Immature-to-Total Neutrophil Ratio (I/T)	Positive	35	0.21 ± 0.03	Mann-Whitney U = 576.0	<0.001**
	Negative	135	0.16 ± 0.03		
Total Leukocyte Count (cells/mm3)	Positive	35	12,484.57 ± 4,369.24	Mann-Whitney U = 2196.0	0.520
	Negative	135	12,852.21 ± 4,319.40		
Platelet Count (cells/mm^3^)	Positive	35	202,285.71 ± 50,589.79	Mann-Whitney U = 2326.5	0.889
	Negative	135	205,111.11 ± 71,423.34		
Absolute Neutrophil Count (ANC)	<1800 cells/mm^3^			χ^2^ = 0.765	0.382
	Positive	35	8 (22.9)		
	Negative	135	41 (30.4)		
	≥1800 cells/mm^3^				
	Positive	35	27 (77.1)		
	Negative	135	94 (69.6)		

Analysis of venous blood parameters revealed marked differences between neonates with positive and negative venous blood cultures (Table 3). Venous CRP levels were substantially higher among culture-positive neonates (19.29 ± 9.37 mg/dL) compared with culture-negative neonates (3.58 ± 2.06 mg/dL; p < 0.001), and the venous I/T ratio was also significantly elevated in the positive culture group (0.21 ± 0.02 vs. 0.16 ± 0.03; p < 0.001). Although the mean venous total leukocyte count was numerically higher among culture-positive neonates, this difference was not statistically significant (p = 0.156). Platelet counts were significantly lower in neonates with positive venous blood cultures compared with those with negative cultures (p < 0.001). Notably, all neonates with positive venous blood cultures had venous ANC values ≥1800 cells/mm^3^, whereas 33 (24.4%) cases of culture-negative neonates exhibited ANC values <1800 cells/mm^3^, resulting in a statistically significant association between venous ANC category and culture positivity (χ^2^ = 10.616, p < 0.001).

**Table 3:** Comparison of Venous Blood Parameters Between Neonates With Positive and Negative Venous Blood Cultures (N = 170) p < 0.01 indicates statistical significance.

Parameter	Venous Blood Culture	n	Mean ± SD/n (%)	Statistical Test	P-value
C-reactive Protein (CRP) (mg/dL)	Positive	35	19.29 ± 9.37	Mann-Whitney U = 72.0	<0.001
	Negative	135	3.58 ± 2.06		
Immature-to-Total Neutrophil (I/T) Ratio	Positive	35	0.21 ± 0.02	Mann-Whitney U = 446.0	<0.001
	Negative	135	0.16 ± 0.03		
Total Leukocyte Count (cells/mm^3^)	Positive	35	14,482.86 ± 2,112.50	Mann-Whitney U = 1995.5	0.156
	Negative	135	13,277.56 ± 4,527.38		
Platelet Count (/µL)	Positive	35	184,000.00 ± 35,496.48	Mann-Whitney U = 1216.0	<0.001
	Negative	135	221,318.52 ± 74,936.85		
Absolute Neutrophil Count (ANC)	<1800 cells/mm^3^			χ^2^ = 10.616	<0.001
	Positive	35	0 (0.0)		
	Negative	135	33 (24.4)		
	≥1800 cells/mm^3^				
	Positive	35	35 (100.0)		
	Negative	135	102 (75.6)		

Based on Youden’s Index, the optimal cutoff value for cord blood CRP to predict neonatal sepsis was 3.3 mg/dL (Youden’s Index: 0.622), while the optimal cutoff for the cord blood I/T ratio was 0.155 (Youden’s Index: 0.630), indicating good discriminatory power. Among the hematological parameters, cord blood platelet count showed limited diagnostic value, with a Youden’s Index of 0.149 at a cutoff of 195000/μL. For venous blood, optimal cutoffs were established at 3.15 mg/dL for CRP and 0.165 for the I/T ratio, with corresponding Youden’s Index values of 0.504 and 0.570, respectively. Venous platelet count showed minimal discriminative ability (Youden’s Index: 0.126 at a cutoff of 110000/μL). A comparison of cord and peripheral venous blood culture results revealed significant concordance (p < 0.001). The detailed diagnostic accuracy of cord and venous blood parameters for predicting neonatal sepsis is shown in Table 4.

**Table 4:** Diagnostic Accuracy of Cord and Venous Blood Parameters for Predicting Neonatal Sepsis (N = 170) CRP: C-reactive protein; I/T ratio: immature-to-total neutrophil ratio; AUC: area under the curve; CI: confidence interval; PPV: positive predictive value; NPV: negative predictive value

Parameter	AUC (95% CI)	Cutoff	Sensitivity (%)	Specificity (%)	PPV (%)	NPV (%)	P-value
Cord Blood							
CRP (mg/dL)	1.000 (1.000-1.000)	3.3	100.0	62.2	48.1	100.0	<0.001
I/T Ratio	0.878 (0.827-0.930)	0.17	100.0	68.9	53.1	100.0	<0.001
Total Platelet Count (per µL)	0.492 (0.388-0.597)	125000	77.1	67.0	22.5	45.5	0.890
Venous Blood							
CRP (mg/dL)	0.985 (0.971-0.998)	3.15	100.0	50.4	41.3	100.0	<0.001
I/T Ratio	0.906 (0.862-0.950)	0.165	100.0	57.0	44.8	100.0	<0.001
Total Platelet Count (per µL)	0.257 (0.171-0.343)	110000	100.0	12.6	28.6	100.0	<0.001

**Figure 1:**
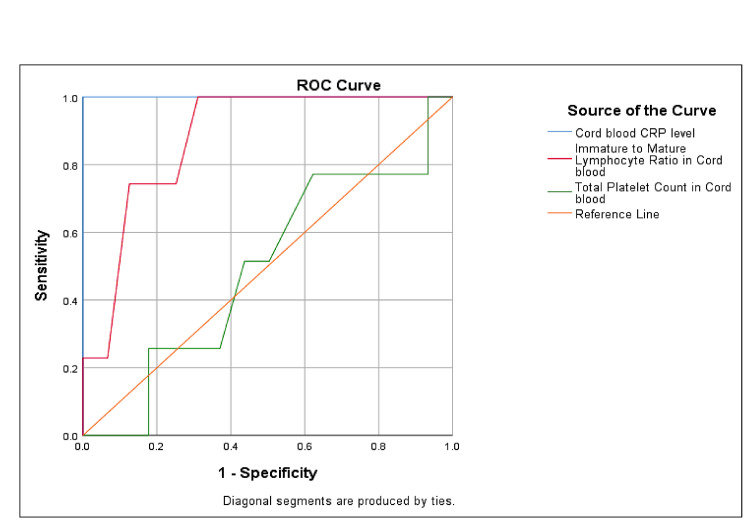
ROC Curve Analysis Results for Cord Blood Parameters ROC: Receiver operating characteristic curve

**Figure 2:**
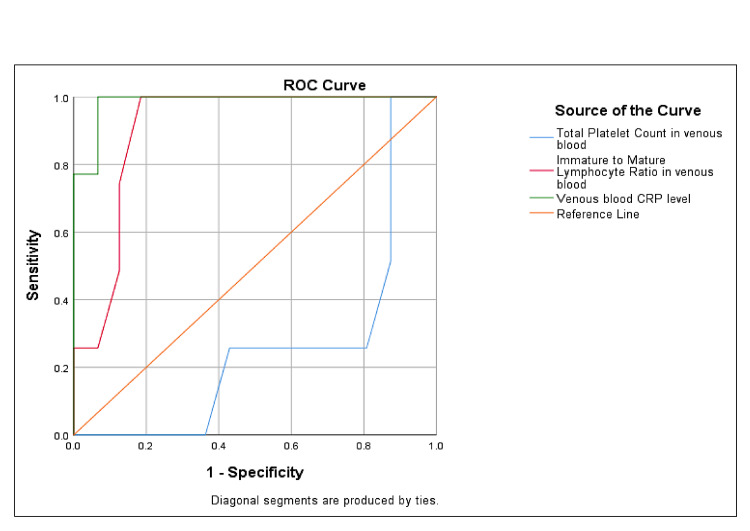
ROC Curve Analysis Results for Venous Blood Markers ROC: receiver operating characteristic curve

ROC analysis was performed to assess the diagnostic accuracy of cord and venous blood biomarkers in predicting EOS. As shown in Figure 1, cord blood CRP exhibited excellent diagnostic performance, with an AUC of 1.000, indicating perfect sensitivity and specificity at an optimal cutoff of 3.3 mg/dL. The cord blood I/T ratio also demonstrated strong predictive ability, with an AUC of 0.878. In contrast, the diagnostic utility of cord blood platelet count was limited, with an AUC close to 0.5, suggesting poor discriminatory power. Similarly, as illustrated in Figure 2, venous blood CRP showed excellent diagnostic accuracy with an AUC of 0.985, while the I/T ratio yielded an AUC of 0.906, both indicating high reliability in detecting culture-positive sepsis. However, venous platelet count again demonstrated limited value, reflected by a low AUC of 0.257.

## Discussion

This study assessed the diagnostic performance of umbilical cord and peripheral venous blood biomarkers for the early identification of neonatal sepsis among newborns exposed to intrapartum maternal risk factors. Cord blood CRP and the I/T ratio emerged as the most reliable early indicators of early-onset sepsis, demonstrating superior discriminative performance compared with other hematological parameters and supporting their potential role in early risk stratification of high-risk neonates. Although the I/T ratio showed strong diagnostic accuracy, transient physiological elevations can occur in healthy neonates immediately after birth due to perinatal stress responses, underscoring the need for interpretation within an integrated clinical and microbiological context. Importantly, cord blood biomarker measurements were obtained solely for research purposes and did not influence real-time clinical decision-making, as antibiotic initiation was guided by standard institutional neonatal sepsis management protocols.

Comparison with existing literature

The diagnostic potential of umbilical cord blood biomarkers, particularly CRP and the I/T ratio, in EOS has been increasingly recognized, although reported performance metrics have shown variability across settings. In this context, our findings add robust evidence by demonstrating exceptionally high diagnostic accuracy for cord blood CRP (AUC 1.000) and I/T ratio (AUC 0.878), with clinically relevant optimal thresholds of 3.3 mg/dL and 0.17, respectively, highlighting their strong discriminative capacity at birth. These results are in strong agreement with the prospective observational study by Srivastav and Bhadoria (2023) [10], who reported 100% sensitivity and specificity for cord blood CRP, along with excellent NPV. Importantly, their study also demonstrated the superior performance of cord-derived CRP over conventional blood culture and peripheral leukocyte indices in neonates exposed to intrapartum risk factors, reinforcing the biological plausibility and clinical relevance of cord blood-based early sepsis screening strategies.

Similarly, Alam et al. (2021) [11] found elevated CRP and I/T ratios in both cord and venous blood among neonates with EOS, reporting perfect sensitivity and high specificity for cord CRP (100% and 89.3%, respectively), closely mirroring our estimates. Their stratification of neonates into proven, probable, and no sepsis categories adds granularity to the analysis and supports the idea that cord blood biomarkers may act as real-time indicators of fetal exposure to infectious or inflammatory stressors. Patrick et al. (2017) [12] similarly confirmed CRP’s diagnostic value, identifying a cord blood threshold of >1.1 mg/L as optimal, with 100% sensitivity and 90.9% specificity. The slightly lower threshold compared to our study may be attributed to their inclusion of neonates without maternal risk factors, leading to a lower pretest probability and hence a more sensitive cutoff. Nonetheless, their conclusions align well with our findings, reinforcing CRP’s potential utility as a universal screening tool.

The study by Annam et al. (2015) [13] also highlighted the utility of the I/T ratio within a cord blood-based hematologic scoring system. They found that an elevated I/T ratio and neutrophil morphological abnormalities were among the most sensitive predictors of sepsis. The inclusion of composite hematologic scores rather than isolated parameters could explain differences in individual marker performance, yet the central role of I/T ratio is consistent with our results. Another study by Meena et al. (2015) [14] found that cord blood CRP was not a reliable indicator of sepsis in their cohort, as all neonates had negative CRP values despite being sepsis screen-positive. This counterintuitive finding may be explained by the delayed onset of systemic inflammation relative to the timing of cord blood sampling or by the inclusion of neonates who had risk factors but no actual microbial invasion.

Kulshrestha et al. (2020) [15] investigated CRP in combination with phosphate and calcium levels and found CRP to be significantly elevated in septic neonates, consistent with our findings. While their primary interest lay in electrolyte imbalances, their work reinforces the systemic inflammatory milieu in EOS and the central role of CRP as a biochemical marker of infection. Chavan and Kshirsagar (2022) [16] demonstrated high diagnostic accuracy of cord blood biomarkers for EOS, identifying PCT as the most sensitive, but still reporting strong ROC performance for CRP and I/T ratio. Their findings underscore the value of combining multiple markers, including CRP and I/T ratio, to enhance diagnostic accuracy, a view that supports the multi-parametric approach adopted in our study.

Strengths and limitations

A key strength of this study lies in its prospective design conducted in a real-world tertiary care setting with standardized protocols for cord blood collection and laboratory analysis. The inclusion of a well-defined high-risk neonatal population enhances clinical applicability, while the use of culture-confirmed sepsis and objective biomarker thresholds strengthens diagnostic validity. Rigorous statistical approaches, including ROC curve analysis and Youden’s Index, were employed to derive optimal cutoff values. Nevertheless, several limitations should be acknowledged. First, this was a single-center study, which may limit the generalizability of the findings to other healthcare settings with differing patient profiles and microbiological patterns. Second, although cord blood sampling was standardized at birth, peripheral venous samples were obtained within a 24-48-hour window based on clinical workflow, which may have introduced some variability in postnatal biomarker kinetics. Third, initiation of antibiotic therapy followed institutional neonatal sepsis management protocols and was guided by clinical assessment rather than study biomarker values; however, early antibiotic exposure in some neonates may have influenced peripheral culture yield and inflammatory marker levels. Finally, a formal concordance analysis between cord blood and peripheral venous culture results was not performed, which limits direct inference regarding microbiological agreement between the two sampling compartments. Future multicenter studies with standardized sampling intervals, longitudinal biomarker assessment, and paired culture concordance analysis are warranted to further validate these findings and enhance external applicability.

Recommendations

Routine assessment of umbilical cord blood CRP and I/T ratio should be considered for neonates born to mothers with intrapartum risk factors, as an early screening approach for exclusion of neonatal sepsis. Future multicentre studies with larger cohorts are needed to validate these findings and to evaluate the incremental value of additional biomarkers, such as PCT or selected interleukins, in refining EOS.

## Conclusions

Umbilical cord blood CRP and I/T ratio appear to be promising, non-invasive biomarkers for early identification of neonatal sepsis among high-risk newborns. At birth, cord blood CRP (cutoff 3.3 mg/dL) and I/T ratio (cutoff 0.17) demonstrated strong diagnostic performance with high sensitivity and NPV, supporting their potential role as adjunctive screening tools to aid early risk stratification and exclusion of early-onset sepsis. However, these biomarkers should be interpreted in conjunction with clinical assessment and microbiological findings. In contrast, cord blood ANC showed limited early diagnostic utility, whereas venous ANC was more strongly associated with established infection, highlighting its complementary value during subsequent clinical evaluation. Further multicenter studies with standardized sampling protocols and longitudinal validation are warranted before routine clinical implementation.

## Appendices

**Table 5:** Study Proforma: Role of Umbilical Cord Blood Sepsis Screening for Prediction of Early-Onset Sepsis in Newborns

Section/Domain	Variable/Parameter	Response/Finding	Remarks/Time point
Section A: Identification & Demographic Details	Date		
	CR Number		
	Serial Number		
	Name of Newborn		
	Father’s Name		
	Sex		
	Birth Weight (g)		
	Gestational Age (weeks)		
	Address		
	Contact Number		
	Date of Admission		
	Date of Birth		
	Time of Birth		
	Duration of Hospital Stay (days)		
	Date of Discharge / Death		
	Outcome (Discharged / Died)		
Section B: Maternal & Perinatal Risk Factors	Spontaneous prematurity (Yes/No)		
	Foul-smelling liquor (Yes/No)		
	Rupture of membranes >24 hours (Yes/No)		
	≥3 sterile vaginal examinations (Yes/No)		
	Prolonged labour (Yes/No)		
	Perinatal asphyxia (Yes/No)		
	Chorioamnionitis (Yes/No)		
	Maternal UTI / genital sepsis (Yes/No)		
	Prenatal fever (Yes/No)		
	Prenatal septicaemia (Yes/No)		
Section C: Anthropometric Parameters	Length (cm)		
	Weight (g)		
	Head Circumference (cm)		
Section D: General Examination at Birth	General condition		
	Temperature (°C)		
	Heart Rate (/min)		
	Respiratory Rate (/min)		
	Blood Pressure (mmHg)		
	Random Blood Sugar (mg/dL)		
	SpO₂ (%)		
	Grunting (Present/Absent)		
	Apnoea (Present/Absent)		
	Chest indrawing (Present/Absent)		
	Capillary refill time >3 sec (Yes/No)		
	Skin pinch >2 sec (Yes/No)		
	Tone		
	Jaundice (Yes/No)		
	Bleeding (Yes/No)		
	Congenital malformation (Specify)		
Section E: Clinical Findings at Admission (Before Antibiotics)	Temperature instability		
	Respiratory system		
	Cardiovascular system		
	Neurological system		
	Gastrointestinal system		
	Umbilical stump		
	Skin changes		
	Joint involvement		
	Bleeding manifestations		
	Other findings		
Section F: Investigations	Hemoglobin (g/dL)		Cord/PV 24h/PV 48h
	Total Leukocyte Count (/mm³)		Cord/PV 24h/PV 48h
	Differential Count		Cord/PV 24h/PV 48h
	Platelet Count (/mm³)		Cord/PV 24h/PV 48h
	I/T Ratio		Cord/PV 24h/PV 48h
	CRP (mg/L)		Cord/PV 24h/PV 48h
	Band cells		Cord/PV 24h/PV 48h
	Blood Culture		Cord/PV 24h/PV 48h

## References

[1] Kariniotaki C, Thomou C, Gkentzi D, Panteris E, Dimitriou G, Hatzidaki E (2024). Neonatal sepsis: a comprehensive review. Antibiotics (Basel).

[2] Li J, Shen L, Qian K (2023). Global, regional, and national incidence and mortality of neonatal sepsis and other neonatal infections, 1990-2019. Front Public Health.

[3] Kumar S, Bhattacharya P, Kaur S, Ray P, Chattopadhyay N (2024). Risk factors and etiology of early-onset neonatal sepsis in northeastern part of India: case-control study. J Family Med Prim Care.

[4] Chawla D (2024). Early-onset neonatal sepsis in India - the ‘elephant’ remains ‘unseen’. Indian J Pediatr.

[5] van Leeuwen LM, Fourie E, van den Brink G, Bekker V, van Houten MA (2024). Diagnostic value of maternal, cord blood and neonatal biomarkers for early-onset sepsis: a systematic review and meta-analysis. Clin Microbiol Infect.

[6] Răcean MA, Săsăran MO, Mărginean CO, Cucerea M (2025). Umbilical cord blood level of interleukins used as a predictor of early-onset neonatal sepsis: a comprehensive review. Front Cell Infect Microbiol.

[7] Yuan J, Wu Y, Zhang Y (2025). Diagnostic value of umbilical cord blood interleukin-6 level in premature infants with early-onset sepsis. Children (Basel).

[8] Kurt AN, Aygun AD, Godekmerdan A, Kurt A, Dogan Y, Yilmaz E (2007). Serum IL-1beta, IL-6, IL-8, and TNF-alpha levels in early diagnosis and management of neonatal sepsis. Mediators Inflamm.

[9] Mithal LB, Becker ME, Ling-Hu T (2025). Cord blood proteomics identifies biomarkers of early-onset neonatal sepsis. JCI Insight.

[10] Srivastav DK, Bhadoria VJS (2023). Role of cord blood CRP and cultures in diagnosis of neonatal sepsis. Int J Adv Res.

[11] Alam MA, Mannan MA, Dey SK, Rahman AZMR, Shahidullah M (2021). Umbilical cord blood for the screening of early onset neonatal sepsis among those at risk of infection. Sch J App Med Sci.

[12] Patrick R, Rajan A, Shriyan A (2017). Cord C-reactive protein as a marker for early onset neonatal sepsis children. Int J Contemp Pediatr.

[13] Annam V, Medarametla V, Chakkirala N (2015). Evaluation of cord blood - haematological scoring system as an early predictive screening method for the detection of early onset neonatal sepsis. J Clin Diagn Res.

[14] Meena J, Charles MV, Ali A, Ramakrishnan S, Gosh S, Seetha KS (2015). Utility of cord blood culture in early onset neonatal sepsis. Australas Med J.

[15] Kulshrestha R, Kulshrestha MR, Kalra RK (2020). The correlation of C-reactive protein and phosphate levels in cord blood samples of neonates with and without sepsis. Sch Int J Obstet Gynecol.

[16] Chavan SS, Kshirsagar VY (2022). Cord blood inflammatory markers for the diagnosis of early-onset neonatal sepsis. Int J Health Sci.

